# Leukocyte-Released Mediators in Response to Both Bacterial and Fungal Infections Trigger IFN Pathways, Independent of IL-1 and TNF-α, in Endothelial Cells

**DOI:** 10.3389/fimmu.2019.02508

**Published:** 2019-10-25

**Authors:** Kieu T. T. Le, Xiaojing Chu, Martin Jaeger, Josée A. Plantinga, Vasiliki Matzaraki, Sebo Withoff, Leo A. B. Joosten, Mihai G. Netea, Cisca Wijmenga, Yang Li, Jill Moser, Vinod Kumar

**Affiliations:** ^1^Department of Genetics, University Medical Center Groningen, University of Groningen, Groningen, Netherlands; ^2^Department of Internal Medicine and Radboud Centre for Infectious Diseases (RCI), Radboud University Medical Center, Nijmegen, Netherlands; ^3^Department of Pathology and Medical Biology, Medical Biology Section, University Medical Center Groningen, University of Groningen, Groningen, Netherlands; ^4^Department of Immunology, K.G. Jebsen Coeliac Disease Research Centre, University of Oslo, Oslo, Norway; ^5^Department of Critical Care, University Medical Center Groningen, University of Groningen, Groningen, Netherlands

**Keywords:** leukocyte-endothelial interaction, sepsis, bacterial infection, fungal infection, leukocyte transcriptomes, endothelial transcriptomes, interferon pathways

## Abstract

In sepsis, dysregulated immune responses to infections cause damage to the host. Previous studies have attempted to capture pathogen-induced leukocyte responses. However, the impact of mediators released after pathogen-leukocyte interaction on endothelial cells, and how endothelial cell responses vary depending on the pathogen-type is lacking. Here, we comprehensively characterized the transcriptomic responses of human leukocytes and endothelial cells to Gram negative-bacteria, Gram positive-bacteria, and fungi. We showed that whole pathogen lysates induced strong activation of leukocytes but not endothelial cells. Interestingly, the common response of leukocytes to various pathogens converges on endothelial activation. By exposing endothelial cells to leukocyte-released mediators, we observed a strong activation of endothelial cells at both transcription and protein levels. By adding IL-1RA and TNF-α antibody in leukocyte-released mediators before exposing to endothelial cells, we identified specific roles for IL-1 and TNF-α in driving the most, but not all, endothelial activation. We also showed for the first time, activation of interferon response by endothelial cells in response to leukocyte-released mediators, independently from IL-1 and TNF-α pathways. Our study therefore, not only provides pathogen-dependent transcriptional changes in leukocytes and endothelial cells during infections, but also reveals a role for IFN, together with IL1 and TNFα signaling, in mediating leukocyte-endothelial interaction in infections.

## Introduction

Sepsis is a life-threatening organ dysfunction caused by a dysregulated host response to infection (1). Despite advances in early recognition, sepsis affects around 30 million people worldwide every year and has a mortality rate of 20–40% (2). Sepsis is known to be a heterogeneous syndrome with various outcomes that depend on pathogenic characteristics as well as on host susceptibility. Despite decades of research, sepsis pathophysiology remains poorly understood. However, it is known that an interaction between innate immune cells and endothelial cells is central for the pathogenesis of sepsis, with recognition of infectious pathogens as a first step toward full activation of inflammation. The activated cells interact with different blood compartments and organ cell types such as platelets, adaptive immune leukocytes, and parenchymal cells (3). Failure in properly regulating these cellular responses and interactions often leads to multiple organ failure, and even mortality in sepsis patients.

Recent studies have employed transcriptomic approaches to characterize the global response of immune cells, particularly peripheral blood mononuclear cells (PBMCs) to various pathogens. After microbial recognition, innate immune cells are prone to inflammatory and stress responses while reducing apoptosis signaling, resulting in the release of cytokines, chemokines, and damage-associated molecular pattern factors into the circulation (4). Gene expression profiles of whole blood from human volunteers challenged with a low dose of endotoxin also showed a reduction in integrin-α and integrin-β chain expression, indicating changes in the host immune system in adherence to and interaction with other cell types (5). Nevertheless, we still know relatively little about the impact of these transcriptomic alterations of immune cells on its interaction with other cell types, specifically on endothelial cells.

Endothelial cells are a monolayer of cells lining the blood vessel that is actively involved in homeostasis as well as interacting with immune cells to regulate inflammation (6). The endothelium is activated not only by inflammatory cytokines such as IL-1β and TNF-α (7), but also by endotoxins such as lipopolysaccharides (LPS) via the Toll like receptor-4 (TLR4) and RIG-I pathways (8, 9). Upon activation, the endothelium secretes cytokines (IL-6 and IL-8) and expresses adhesion molecules (E-selectin, VCAM-1, and ICAM-1) to facilitate leukocyte extravasation. The increased influx of neutrophils and monocytes into the tissue during sepsis could then indirectly lead to tissue damage by secreting exaggerated amounts of inflammatory mediators and reactive molecules (10). In the context of sepsis, the endothelium can be exposed to different types of infectious pathogens such as bacteria (*Streptococcus pneumoniae*) or fungi (*Candida albicans*). However, the impact of these pathogens on endothelial activation has not been studied systematically.

Given the ability of endothelial cells to respond to pathogens and interact with immune cells, it is important to characterize endothelial responses upon exposure to the mixture of various cytokines, chemokines and proteins secreted by activated immune cells, as well as to different types of infectious pathogens. Therefore, in this study, we applied a two-step *in vitro* stimulation model to comprehensively characterize: (1) the transcriptomic responses and inflammatory proteins secreted by PBMCs in response to a variety of stimulating pathogens, including Gram-negative bacteria, Gram-positive bacteria, and fungi; and (2) the transcriptomic responses of endothelial cells exposed to humoral signals from activated PBMCs that were exposed to various pathogens. Through this work, we were able to identify the role of IL-1 and TNF-α in driving most, but not all, endothelial activation. We show that, independent of IL-1 and TNF-α, interferon (IFN) pathways in endothelial cells are strongly induced by humoral signals from activated leukocytes.

Our study provides crucial insights into the role of pathways mediating leukocyte-endothelial interactions, including IL-1, TNF-α, and IFN pathways. Further studies are required to validate the function of IFN pathways in endothelial function and IFN's role in determining sepsis progression.

## Materials and Methods

### PBMC Isolation

Venous blood samples were collected from healthy volunteers. All donors provided written informed consent. Ethical permission for this study was approved by the Ethical Committee of Radboud University Nijmegen (nr 42561.091.12). Blood was collected in EDTA tubes (BD vacutainer). PBMCs were quickly isolated within 3 h of collection. Blood was diluted with 1 volume of DPBS (Gibco, ThermoFisher Scientific) before adding to Ficoll-Paque (Pharmacia Biotech). Gradient centrifugation was performed for 30 min at 400 g, using no brake. After centrifugation, the layer containing PBMCs was collected using a Pasteur pipette. PBMCs were washed twice with PBS, counted (BioRad cell counter), and adjusted to reach the final concentration of 2 million cells/ml in RPMI 1640 (Gibco, ThermoFisher Scientific), supplemented with 10% heat-inactivated Fetal Cow Serum (Gibco, ThermoFisher Scientific), gentamicin 10 mg/ml, L-glutamine 10 mM, and pyruvate 10 mM. Cells were seeded into wells to settle overnight before stimulation.

### PBMC Stimulation

To study PBMC transcriptomes upon five types of heat-killed pathogens, PBMCs were stimulated with various pathogens, including heat-killed *Streptococcus pneumonia* (ATCC 49619, serotype 19F) at 1 million cells/ml, heat-killed *C. albicans* (ATCC MYA-3573, UC 820) at 1 million cells/ml, heat-*killed Aspergillus fumigatus* (V05-27) at 1 million cells/ml, *Mycobacterium tuberculosis* (H37Rv) at 1 million cells/ml, and heat-killed *Pseudomonas aeruginosa* at 1 million cells/ml (11). Cells were also incubated with RPMI 1640 only as a negative control. RNA was isolated from PBMCs at 4 and 24 h after stimulation.

### Endothelial Cell Culture and Direct Stimulation

Primary Human Umbilical Vein Endothelial Cells (HUVECs) were used to study the response of endothelial cells upon infection. Pooled donor HUVECs were purchased (Lonza, Breda, the Netherlands) and cultured in EBM-2™ medium (Lonza) supplemented with EGM-2 MV SingleQuot Kit Supplements & Growth Factors (Lonza) at 37°C, 5% CO_2_ and saturating humidity. Passage 3–5, confluent cells were used for all experiments.

For direct stimulation, HUVECs were stimulated with either heat-killed *Streptococcus pneumonia (*ATCC 49619, serotype 19F) at the concentration of 1 million cells/ml, heat-killed *C. albicans (*ATCC MYA-3573, UC 820) at 1 million cells/ml, LPS (*Escherichia coli* serotype O26:B6, Sigma, St. Louis, MO, USA) at 1,000 ng/ml, IL-1β (Biosource Netherlands, Etten-Leur, The Netherlands) at 10 ng/ml, TNF-α (Biosource Netherlands) at 10 ng/ml for 6 or 24 h.

### Leukocyte-Endothelial Cell Interaction

To study the effect of soluble factors released by activated PBMCs on endothelial cells, PBMCs were diluted to 2 million cells/ml and stimulated with three different types of pathogens at the ratio of 2 cells:1 pathogen heat-killed *Streptococcus pneumonia*, heat-killed *C. albicans* and LPS (10 ng/ml). RPMI medium was used as the negative control. Supernatants were collected after 24 h of stimulation, aliquoted, filtered (0.45 μm filter) and kept at −20°C before either exposing to HUVECs or measuring cytokine levels with OLINK and ELISA.

The supernatants from activated PBMCs were thawed overnight at 4°C and warmed up shortly to 37°C. Polymyxin B (InvivoGen, Toulouse, France) was added to the supernatants at a final concentration of 100 μg/ml to neutralize residual LPS (9). The supernatants were then added to HUVECs. To study the effect of IL-1 and TNF-α secreted by activated PBMCs on endothelial cells, LEAF-purified TNF-α Antibody (BioLegend, San Diego, CA, USA) and/or IL-1RA (Anakinra) were added to the supernatants at the final concentration of 4 and 300 ng/ml, respectively, incubated at 37°C for 1 h before adding to HUVECs (https://patents.google.com/patent/US7227003). HUVECs were incubated with the supernatants from stimulated PBMCs for 6 and 24 h. At the time of harvesting, conditioned medium, and cells were collected for ELISA, RNA isolation and flow cytometry.

### RNA Isolation

Cells were harvested and lysed in lysis buffer from the MirVanva MagMax RNA isolation kit Applied Biosystems Nieuwerkerk aan den IJssel, The Netherlands. RNA was isolated according to the manufactures instructions. RNA concentration was measured based on Optical density (OD260) using the Nanodrop machine (NanoDrop Technologies, Rockland, ME, USA). RNA integrity was determined using the Bioanalyzer (Agilent D2000). All samples had RIN score > 9.

### RNA Sequencing and Pathway Enrichment Analysis

For PBMC sequencing, 1,000 ng of total RNA (RIN score ≥ 9) were submitted for RNA library preparation using the NEXTflex TM Rapid Directional RNA-seq kit, BioScientific. NGS libraries were enriched for polyA tail RNA. Samples were sequenced using the Illumina NextSeq 500 platform, single-end read. Samples were randomly assigned into different flows and sequenced to reach 12–15 million reads per sample. Sequencing reads were then mapped to the human genome using STAR (version 2.3.0) with a reference to Ensembl GRCh37.71. Read counts per gene was quantified by Htseq-count, Python package HTseq (version 0.5.4p3) using the default union-counting mode (*The HTSeq package*, http://htseq.readthedocs.io/). For endothelial RNA sequencing. 500 ng of total RNA (RIN score ≥ 9) were sent to GenomeScan, Leiden, The Netherlands for analysis. mRNA (polyA) enriched libraries were constructed, and sequenced with the NextSeq 500 platform, single end, 75 bp with 15–20 million reads/ sample. Fragments were aligned using Hisat2. Raw counts were calculated with String Tie. Pathway analysis was performed using gene set enrichment analysis on differential expressed genes using the default setting from ConsensusPathDB-human database (http://cpdb.molgen.mpg.de).

### Flow Cytometry

To determine the protein expression of adhesion molecules on the HUVEC membrane, cells were washed with PBS, detached using trypsin, washed with PBS, and re-suspended in ice-cold FACS buffer (PBS supplemented with 5% FCS). The cells were divided equally into separate FACS tubes. The cells were stained using the following antibodies: PE-conjugated anti-human E-selectin (CD62E) (Biolegend), APC-anti human VCAM-1 (CD106) (Biolegend), FITC- anti human ICAM-1 (CD54) (Biolegend), and IgG isotope controls (IgG isotope controls, Biolegend) for 30 min on ice. The cells were washed once and resuspended in FACS buffer. Samples were analyzed using a MACSQuant Analyzer 10 system (Miltenyi Biotech, San Diego, CA, USA). Multi-color compensation was calibrated using positive control cell population (LPS activated HUVECs). Data were presented as the Geometric Mean of Fluorescence Intensity (MFI).

### ELISA

Cytokine levels secreted by PBMCs and/or HUVECs were determined using the ELISA Duo kits, IL-6 (R&D), IL-1β (R&D), IL-1α (R&D), TNF-α (R&D) and IL-8 (R&D), according to the manufacturer's instructions. Data were presented as pg/ml.

### OLINK

To further quantify the levels of other secreted proteins upon various stimulations, supernatant samples were analyzed by proximity extension assay provided commercially by Proseek Multiplex analysis (Olink Bioscience, Uppsala, Sweden) using their inflammation panel (https://www.olink.com/products/inflammation). In brief, for each marker, a pair of nucleotide probe-conjugated antibodies was incubated with the sample. Only when binding to target antigen presented in the sample, the pair of probes are in proximity, enabling the probes to anneal and amplify during Realtime PCR. Internal control was used to minimize variation within runs. The output data is an arbitrary unit of normalized log_2_ expression scale (NPX- normalized protein expression). The NPX value is different for each protein due to the sensitivity of each of the probes. The range and estimated inversion from NPX value to absolute amount (ng/μl) can be found in Olink website. Data were shown as NPX value.

### Statistical Methods

For RNAsequencing data, differentially expressed genes were identified by statistical analysis using the DESeq2 package from Bioconductor. A statistically significant threshold (FDR P ≤ 0.05 and fold change ≥ 2) was applied. For pathway analysis, significant threshold (FDR <= 0.05) was used to identify significant pathways. For FACS and ELISA, graphs and statistical tests were performed using GraphPad Prism software v.6 (GraphPad Prism Software Inc., San Diego, CA, USA). Differences were considered significant when *p* < 0.05. ELISA and FACS data were checked for normality distribution with Omnibus K2 test. One-way ANOVA with Turkey multiple comparison tests were performed to identify significant differences between conditions and control for direct stimulation.

## Results

### Pathogen-Dependent Early and Late Transcriptional Responses of PBMCs

**Figure 1:**
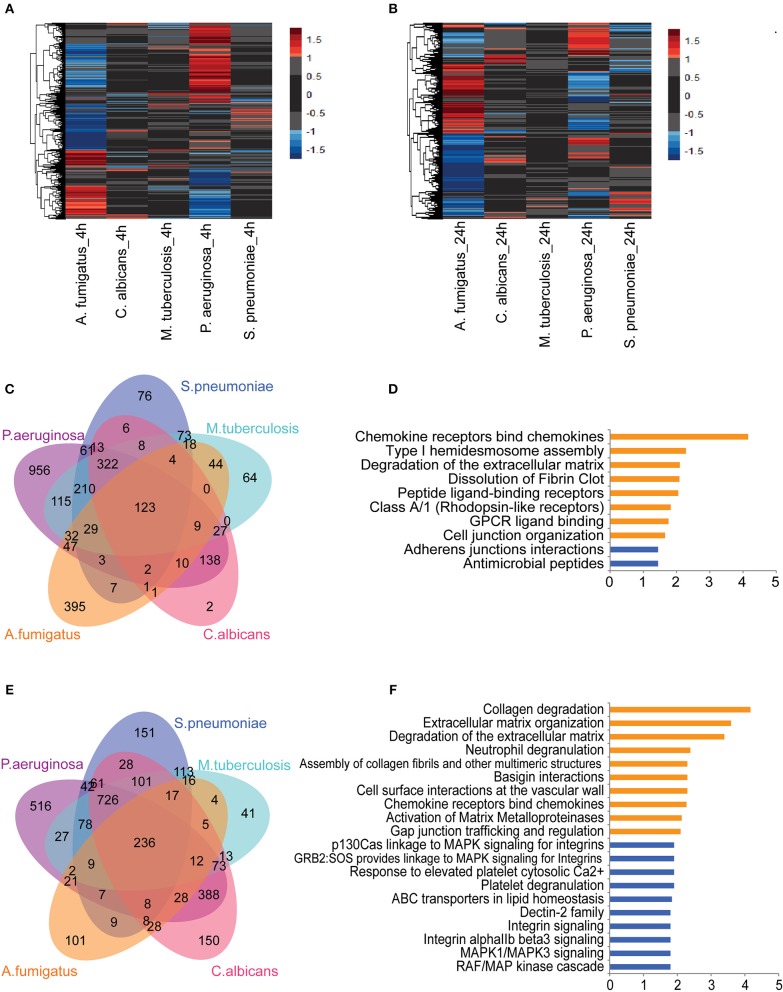
Core transcriptional responses of PBMC to different stimulations affect EC. The expression levels (log_2_-fold change) of differentially expressed (DE) genes in PBMC upon stimulation at **(A)** 4 h and **(B)** 24 h. Color represents log_2_-fold change value. Number of shared and specific DE genes at **(C)** 4 h and **(E)** 24 h. Pathways enriched for common DE genes at **(D)** 4 h and top-10 pathways enriched at **(F)** 24 h. Orange and blue indicate pathways enriched by upregulated and suppressed genes, respectively. Data are represented as **(A,B)** mean expression levels from PBMC isolated from eight individuals **(D,F)** –log_10_ *q*-value.

To identify pathogen-dependent transcriptional responses in leukocytes, we first studied the global transcriptional changes of human PBMCs upon various stimuli. PBMCs were isolated from eight healthy individuals and stimulated by five types of pathogens: *Pseudomonas aeruginosa* (*P. aeruginosa*), *Streptococcus pneumoniae* (*S. pneumonia*)*, Mycobacterium tuberculosis* (*M. tuberculosis*), *Candida albicans* (*C. albicans*), and *Aspergilus Fumigatus* (*A. fumigatus*) for 4 and 24 h. We performed RNA sequencing followed by differential expression analysis to identify differentially expressed (DE) genes between stimulated and un-stimulated samples (RPMI control). This identified 4,189 protein-coding genes that were significantly differentially regulated in response to at least one of the stimulations (Adjusted *P* ≤ 0.05, FC ≥ 2-fold). Among those DE protein-coding genes, we observed both common genes, which respond to all pathogens, and pathogen-specific genes at 4 h (Figure 1A) and 24 h of stimulation (Figure 1B). We observed that the Gram-negative bacteria *P. aeruginosa* altered the expression levels of more than 2,000 genes at 4 h. In contrast, we found fewer genes to be differently regulated in response to *C. albicans* (666 genes) and Gram-positive bacteria (956 genes) at 4 h (Supplemental Figure S1A). At 24 h, we found more genes being differentially regulated by different pathogens. This indicates that *P. aeruginosa* is one of the stronger inducers of early responses in leukocytes (Supplemental Figure S1B). In contrast, *C. albicans* induced three times more genes, indicating it is a strong immune stimulator at later time points.

Next we performed pathway enrichment analyses on pathogen-specific DE genes (Supplemental Table S1). Pathway enrichment analysis of *P. aeruginosa*-specific genes at 4 h showed significant enrichment of DE genes for several immune pathways, including cytokine responses, IFN signaling, TNF signaling, IL-1 signaling, apoptosis, and inflammasome activation. Interestingly, *S. pneumonia*-specific DE genes are enriched for the suppression of inflammatory pathways and TCR signaling at both 4 and 24 h. In contrast, *C. albicans* specific pathways are enriched for antigen presentation and initiating inflammatory responses (Supplemental Table S1). Overall, the distinct pathways enriched with DE genes by each pathogen highlight the induction of different inflammatory responses in PBMCs: TNF signaling, IL-1 signaling, and IFN signaling. The transcriptome response, thus reflects complex cytokine responses of PBMCs that are needed to interact with different cell types, depending on the type of infectious pathogens.

### Common Responses of PBMCs to a Variety of Pathogens Converge on Endothelial Cells

Next we tested whether common genes that are differentially regulated in response to all pathogens are enriched for particular pathways. At 4 h of stimulation, there were 123 genes that responded to all five pathogens (Figure 1C). Of note, some of the pathways that were activated at the early time point (4 h) also remained active at 24 h. Among 123 common genes that are either induced or repressed at 4 h, 50% show consistent differences at 24 h. Interestingly, chemokine genes such as *CCL2, CCL3, CCL7, CXCL8* (IL-8), and IL-10 are more strongly induced at 24 h, indicating the role of chemokine signaling in communicating with different cell types at later time points (Supplemental Figure S2). Pathway enrichment of the 123 common genes showed the enrichment of genes involved in initiating chemokine responses as well as in arranging cell-cell interaction, and assembly of cell junctions. Interestingly, expression levels of the cadherin genes *CDH5* and *CDH6* are reduced, suggesting a repression of adherens junction interactions (Figure 1D). At 24 h, we found 236 DE genes shared between all pathogens (Figure 1E). The up-regulated genes were enriched for the interaction of immune cells with the extracellular matrix and vascular cell wall, as well as for regulation of trafficking through gap junction (Figure 1F). These results show that the common pathways induced in leukocytes in response to different sepsis-causing pathogens are also involved in regulating the interaction of immune cells with the cellular matrix and in interaction with endothelial cells at the vasculature. Endothelial cells are known as a non-classical innate immune cell type that recognize and respond to bacterial lipopeptides via Toll-like receptor signaling. Endothelial responses to infection produce cytokines and chemokines, alter leukocyte migration, facilitate coagulation and, ultimately, contribute to controlling infection (12). Therefore, based on the complexity of cytokine signals released by leukocytes to different types of infection and their convergent effect on endothelial cells, it is crucial to understand the impact of leukocyte humoral signals on endothelial cells and their coordination to fight against infections.

### Minimal Impact of Heat-Killed Pathogens on Endothelial Inflammatory Responses

**Figure 2:**
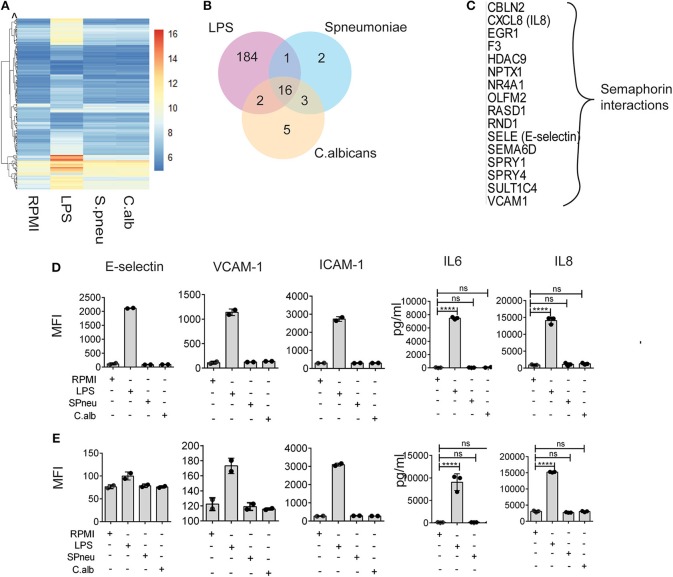
Heat-killed pathogens do not directly induce EC activation. **(A)** Mean RNA expression level (VST) of 213 DE genes responding to either LPS, *S. pneumoniae* or *C. albicans* on EC. Color scales by VST value, ranging from weak to strong expression (6–16). Data are represented as mean of three replications. **(B)** Number of shared and unique genes induced in EC upon stimulation. **(C)** List of 16 common DE genes (with protein names), and their pathway enriched by Reactome. **(D,E)** Protein expression levels of E-selectin, VCAM-1, ICAM-1, IL-6, and IL-8 in EC at 6 and 24 h after stimulation with LPS, *S. pneumoniae (SPneu)* and *C. albicans (C.alb)*, respectively. E-selectin, VCAM-1 and ICAM-1 were measured by flow cytometry, data were presented as Geometric Mean Florescence Intensity (MFI). IL-6 and IL-8 were measured by ELISA, data were presented as pg/ml. Data are shown as mean (*SD*), representative for three independent experiments. ****p < 0.0001.

In sepsis, endothelial barrier disruption is commonly observed in septic shock where organ function fails (13). However, not much is known about whether different types of pathogens can activate vascular endothelial cells directly. We therefore investigated if HUVECs can respond to direct stimulation by LPS, heat-killed *S. pneumoniae* or heat-killed *C. albicans*. Transcriptome profiles of HUVECs after 6 h direct stimulation showed strong response of HUVECs to LPS in contrast to heat-killed pathogens (Figures 2A,B). We found 203 genes that significantly responded to LPS, whereas only 22 and 26 genes were induced by *S. pneumoniae* and *C. albicans*, respectively (Figure 2B). There were 16 genes in HUVECs that responded to all stimulations (Figure 2C), and these were enriched for alterations in semaphorin interaction. RNAseq data also showed significant differences in the expression levels upon stimulation of the inflammatory markers: IL-8 (*CXCL8*), E-selectin (*SELE*), and VCAM-1 (*VCAM1*). However, the small changes in RNA levels of these markers upon *S. pneumonie and C.albicans* stimulation did not significantly alter protein levels (Figures 2D,E). Therefore, direct activation of endothelial cells by pathogens has minimal effect on inducing endothelial cell inflammatory responses. We therefore hypothesized that in response to pathogens, the leukocytes produce mediators that activate endothelial cells much more strongly than direct pathogen stimulation.

### Leukocyte-Released Mediators Significantly Induce Endothelial Cell Activation

**Figure 3:**
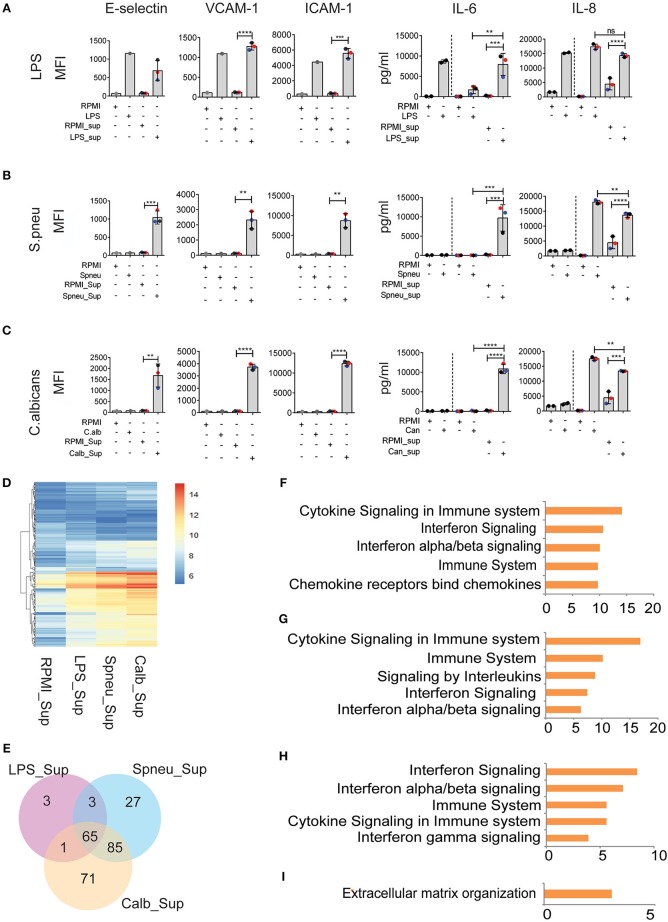
Leukocyte-mediators significantly induce EC activation. **(A–C)** Protein levels of E-selectin, VCAM-1, and ICAM-1 measured by flow cytometry, and secreted IL-6 and IL-8 measured by ELISA on EC after 6 h exposed to direct stimulation (first 2 columns) and to PBMC medium (other columns). Data were presented as Mean florescence Intensity (MFI) for E-selectin, VCAM-1 and ICAM-1 and pg/ml for IL-6 and IL-8. For IL-6 and IL-8, the amount of cytokines presented in PBMC medium before adding to EC were plotted in the 3rd and 4th column, whereas the amount of total cytokines after exposure to EC were plotted in the 5th and 6th column. Colors represent three different individuals of whom PBMC were isolated. **(D)** RNA expression levels (VST) of 255 DE genes in EC induced by PBMC medium. **(E)** The number of shared and unique DE genes between various conditions. **(F)** Top five pathways enriched by 60 common genes induced by all PBMC medium. **(G–I)** Top 5 pathways enriched by genes responded to **(G)** Spneu_Sup and Calb_Sup, **(H)** only Calb_Sup, and **(I)** only Spneu_Sup. Data are shown as **(A–C)** representative of three independent experiments (mean and *SD*) **(D,E)** mean from three biological replications **(F–I)**, –log_10_ of *q*-value. **p < 0.01, ***p < 0.001, ****p < 0.0001.

During bloodstream infection, endothelial cells are in contact with both infectious pathogens and immune cells. RNAseq data from activated PBMCs indicated up-regulation of several cytokine pathways upon stimulation with different types of pathogens. We therefore investigated the effect of cytokines from stimulated immune cells on endothelial cells. To mimic the humoral interaction between immune cells and endothelial cells, we first stimulated PBMCs with either LPS, *S. pneumoniae* or *C. albicans* for 24 h, then harvested the supernatant, neutralized the LPS-trace by polymyxin B (9), and exposed HUVECs to the supernatant containing cytokine signals from the activated PBMCs. After 6 h of exposure, we measured the expression levels of inflammatory markers on endothelial cells. We observed strong activation of endothelial cells at protein level. Interestingly, whereas direct exposure of HUVECs to heat-killed pathogens did not induce endothelial activation, the exposure to supernatants from *S*. *pneumonia*-stimulated PBMCs (Spneu_Sup) and *C. albicans*-stimulated PBMCs (C.alb_Sup) significantly induced the expression levels of adhesion molecules (E-selectin, VCAM-1, and ICAM-1) and cytokines (IL-6 and IL-8) (Figures 3A–C). Endothelial cells, together with PBMCs, are the main source of IL-6. We conclude that endothelial cells become activated by heat-killed pathogens via cytokines and other inflammatory mediators released from activated PBMCs.

To further characterize the genes and pathways activated in HUVECs outside the conventional markers, we performed RNAseq to look at the transcriptome of endothelial cells exposed to PBMC supernatants (Figures 3D,E). We found 72 genes induced by LPS_Sup, 180 genes induced by Spneu_Sup and 222 genes induced by Calb_Sup. Among these, 65 genes are shared between all supernatants, which is 90% of the LPS_Sup responding genes, 36% of Spneu_Sup responding genes, and 29% of Calb_Sup responding genes (Figure 3E). Among the 65 shared genes, 60 are uniquely induced by supernatant and five are also commonly induced by direct stimulation (*CXCL8, F3, RND1, SELE, VCAM1*). Pathway enrichment for the 60 unique genes shows a strong enrichment for cytokine signaling, IFN signaling, IL-1 signaling and TNF signaling (Figure 3F and Supplemental Figure S3). Since LPS, hence LPS_Sup, cannot represent the complexity of PBMC responses to bacteria, we also looked at the genes shared between Spneu_Sup and Calb_Sup. Interestingly, here we found 85 commonly responding genes, which is 47% of the S.pneu_Sup and 38% of the Calb_Sup responding genes. Pathway enrichment for these genes also indicated strong enrichment for cytokine signaling, particularly for interleukins, IFN, and IL-1 (Figures 3G–I). Altogether, this evidence suggests that the activation of endothelial cells by mediators released from PBMCs is mostly shared and independent of the type of infectious pathogens.

### IL-1 and TNF-α Are Major Mediators, Yet There Are Contributions From Other Cytokines Secreted by PBMCs on EC Activation

**Figure 4:**
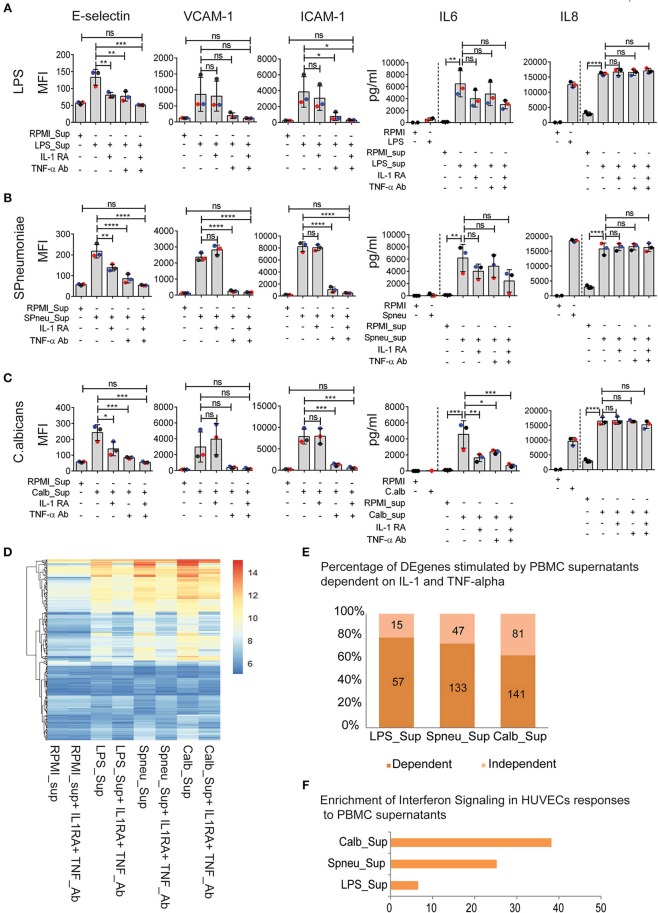
IFN pathways remain active in EC after neutralization of IL1 and TNFα in leukocyte-mediators. **(A–C)** Protein abundance on EC after exposure to **(A)** LPS-activated PBMC medium **(B)**, *S. pneumonia*-activated PBMC medium **(C)**, *C. albicans*-activated PBMC medium with, or without IL-1RA and TNF-α Ab for 6 h. For cytokines, the amount of cytokines presented in PBMC medium before adding to HUVECs were plotted before the dash line whereas that after 6 h exposed to HUVECs were plotted behind the dash line. Colors of dots indicate different PBMC donors. **(D)** RNA expression levels (VST) of 255 DE genes activated in EC by PBMC medium with or without blocking. **(E)** Percentage of genes independent from IL-1 and TNF-α. Number of genes that are dependent (dark shade) and independent (light shade) of IL1 and TNF α. **(F)** Genes expressed independent of IL-1 and TNF-α are strongly enriched for IFN signaling. Data are shown as **(A–C)** mean (*SD*), representative of three independent experiments **(D)**, mean expression value of 3 biological replications **(F)**, –log_10_ of *q*-value. *p < 0.05, **p < 0.01, ***p < 0.001, ****p < 0.0001.

Although blocking of IL-1 or TNF-α has resulted in inconsistent results due to study design, recent clinical trials in stratified patients have shown IL-1- or TNF-blocking therapy to be effective in improving sepsis survival (14–16). Since endothelial cells express receptors for IL-1 (17, 18) and TNF-α (19), we were intrigued to investigate the effect of IL-1 and TNF-α present in the PBMC-supernatant on inducing endothelial activation, and if other PBMC secreted-mediators play a role. Before adding the supernatants to HUVECs, we either neutralized TNF-α in the supernatants with a TNF-α blocking antibody or blocked the effect of IL-1α and IL-1β by adding IL-1RA (Anakinra), or both. The blocking dose efficiency was 100% for IL-1 and approximately 75% for TNF-α (Supplemental Figure S3). We found that TNF-α secreted by activated PBMCs was the main mediator for endothelial expression of adhesion molecules (E-selectin, VCAM-1, and ICAM-1). However, it was not the sole mediator. IL-1α and/or IL-1β also activated the expression of E-selectin, but not VCAM-1 and ICAM-1 (Figures 4A–C). Moreover, neutralization of both TNF-α and IL-1 in the supernatant secreted by activated PBMCs almost completely inhibited endothelial activation, indicating an additive effect of TNF-α and IL-1 on endothelial cell activation (Figures 4A–C). On the other hand, TNF-α had no effect on the induction of IL-6 secretion by endothelial cells. This is in contrast to IL-1 blockage, which inhibited endothelial IL-6 secretion. However, the extent to which IL-1 regulates IL-6 expression on endothelial cells depends on other mediators that are co-secreted by PBMCs in response to a specific pathogen. Neutralization of IL-1 in the PBMC supernatant reduced the amount of IL-6 secretion only in the case of *C. albicans*. Of note, cytokine levels secreted by endothelial cells do not completely return to baseline levels even after blocking TNF-a and IL-1, which suggests that other pathways may be involved in producing endothelial cytokines at a marginal level.

### Up-Regulation of IFN Pathways in Endothelial Cells by Humoral Mediators From PBMCs Is Independent of IL-1 and TNF-α

As IL-1 and TNF-α are the major mediators that induce a strong response in endothelial cells, we tested the effect of blocking IL-1 and TNF-α on endothelial transcriptional responses (Figures 4D–F). Comparison of DE genes in HUVECs exposed to supernatants before and after TNF-α Ab and IL-1RA treatment revealed differential expression of 15, 47, and 81 genes in the context of LPS, *S. pneumoniae* and *C. albicans*, respectively (Figure 4E). Interestingly, these genes were shared between different supernatants and are enriched for IFN-α/β and IFN-γ pathways (Figure 4F). The enrichment was much stronger in the case of *C. albicans* stimulation suggesting *C. albicans* is one of the strong stimulators of IFN-inducing mediators. Notably, in the context of *S. pneumoniae* and *C. albicans*, we found the differential expression both IFN-induced genes and the upstream genes, including *DDX58* (RIG-I), *NLRC5*, and *TLR3*. We also found the expression of IFN receptor genes in HUVECs at RNA levels (Supplemental Figure S4A). These results suggest that the IFN-α/β and IFN-γ pathways are up-regulated in endothelial cells by mediators released by leukocytes in response to sepsis-causing pathogens, and are independent on IL-1 and TNF-α.

### Validation of an IL-1- and TNF-α-Independent Effect of IFN-α/β/γ on Endothelial Response

**Figure 5:**
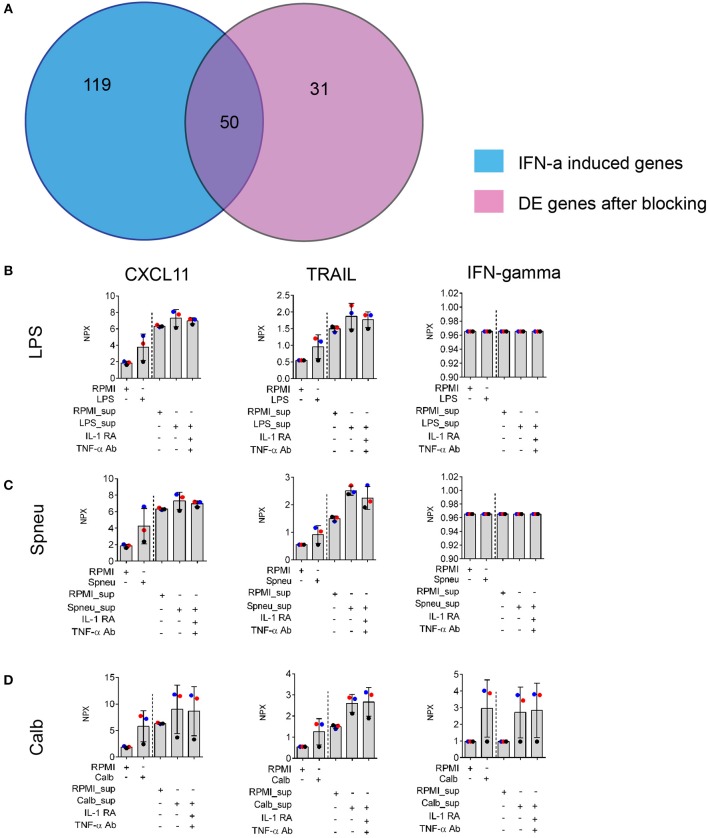
Validation of IFNs effect on EC. **(A)** Number share and unique genes induced by IFN-α on ECs (20) and leukocyte- mediators after blocking of both IL-1 and TNF-α on ECs. **(B–D)** Protein levels of IFN-α downstream genes: CXCL11 and TRAIL, and IFN-gamma produced by stimulated leukocytes (before the dash line) and EC (after the dash line) in the supernatants, measured by Olink®. Data are shown as mean (SD), represented for two independent experiments. Colors correlate to three donors of whom PBMCs were isolated.

In view of the above data, to test if IL-1- and TNF-α-independent effect on endothelial cells was mainly driven by IFN-α/β/γ, we made use of previously published microarray gene expression data (20). We compared whether the genes induced by direct activation of HUVEC by IFN-α/β/γ are also induced in our experiment after blocking IL-1 and TNF-α. We found that 62% of genes induced in HUVECs after exposure to *Candida*-induced supernatant with IL-1 and TNF-α blocking agents are also present in the list of differential expressed genes induced by IFN-α (Figure 5A). We also found expression of IFN-β and IFN-⋎ in PBMCs in response to stimulation at RNA levels (Supplemental Figure S4B). Altogether, it suggests the presence of IFN-α in the mediators released by leukocytes. We also found high levels of circulatory IFN-γ in the supernatants from *Candida*-stimulated leukocytes (Figure 5D). In addition, we also confirmed the up-regulation chemokines such as CXCL11 and TRAIL which is known to be present at higher levels in the endothelium following treatment with IFN-α (20). Of notice, protein levels of these proteins are not dependent on IL-1 and TNF-α (Figures 5B–D).

## Discussion

Sepsis is not a homogeneous disease, but rather life-threatening organ dysfunction syndrome caused by a dysregulation of host response to infection (1). Given the heterogeneity of sepsis patients, many clinical trials targeting the hyper-inflammatory response in sepsis, including corticosteroids, and anti-cytokine therapies (e.g., anti-IL1 and anti TNF-α) have yielded disappointing results (21, 22). Understanding the impact of the complex interactions between causal cell types in a pathogen-specific manner is therefore crucial to delineate the molecular basis of heterogeneity in sepsis outcome. In the present study, we applied an integrative genomics approach to not only characterize the global transcriptional response of leukocytes and endothelial cells to many sepsis-causing pathogens, but to also identify important molecular pathways induced during leukocyte-endothelium cross-talk in regulating overall immune response in sepsis.

First, to identify leukocyte responses to three classes of infectious pathogens (Gram-negative bacteria, Gram-positive bacteria, and fungi) we assessed the transcriptional response of PBMCs to *P. aeruginosa, S. pneumoniae, M. tuberculosis, C. albicans*, and *A. fumigatus*. This allowed us to identify several pathways that were induced in a pathogen-specific manner. The *S. pneumoniae*-specific transcriptome revealed the repression of genes involved in IFN signaling and antigen presentation, while *P. aeruginosa*-specific genes were enriched for the TNF-α and cytokine signaling pathways. These findings suggest that pathogen-specific responses in leukocytes could influence pathophysiology found sepsis patients, partly underlying the heterogeneity observed in sepsis. However, our analysis also identified core pathways that were induced in PBMCs in response to all pathogens as reported before (4). This suggests that the common genes that correspond to core pathways such as antigen presentation, cell-cell signaling, immune regulatory pathways, are absolutely necessary to fight against all types of bacterial or fungal infections. Importantly, we found a strong enrichment of genes involved in leukocyte interaction with endothelial cells and cellular matrix. This finding suggests that the core leukocyte response to pathogens converges on endothelial cells. Interestingly, as suggested by our previous study (23), genes located nearby genetic variants that are associated with sepsis are enriched for immune and endothelial pathways. Therefore, studying leukocyte-endothelium cross-talk is critical in the context of sepsis and potentially can explain sepsis heterogeneity.

Second, by characterizing the global transcriptional response of endothelial cells to sepsis causing pathogens, we emphasize the role of leukocyte-endothelial cross-talk during infections. Although endothelial cells are not considered classical immune cells, HUVECs have been shown to express TLR4 and RIG-I, pattern recognition receptors for LPS-mediating responses to regulate cytokine responses and endothelial activation (8, 9). We indeed observed very strong activation of endothelial transcriptional pathways in response to LPS in comparison to direct stimulation of endothelial cells using other bacterial and fungal pathogens. These findings are in line with the observation of Filler et al. who showed that heat-killed *C. albicans* were not sufficient to induce strong expression of cytokines and adhesion molecules in endothelial cells in comparison to live and germinating *C. albicans* (24). Our study mapped the whole transcriptomic response to various pathogens including heat-killed *S. pneumoniae* and heat-killed *C. albicans*, which to our understanding, has not been reported before. Moreover, we showed that endothelial responses to Gram-positive bacteria and fungi are strongly affected by inflammatory signals from activated leukocytes. More than 66% of responding genes in HUVECs stimulated by PBMC humoral signals are shared across all stimuli. Although we expected to find activation of endothelial cells in response to circulatory mediators released by leukocytes, it was interesting to find a strong induction of IFN pathways together with IL-1 and TNF-α pathways. Whereas, IL-1 and TNF-α pathways have been investigated as a strategy to improve sepsis outcome, by studying the impact of humoral signals from leukocytes on endothelial cells, we observed a stronger enrichment of IFN-α/β and IFN-γ signaling than IL-1 and TNF-α signaling in endothelial cells. This suggests that, together with IL-1 and TNF-α, IFN pathways can result in aberrant responses within endothelial cells. Moreover, by neutralizing IL-1 with Anakinra and TNF-α with TNF-α blocking antibody in leukocyte humoral signals, we confirmed that IL-1 and TNF-α are the major mediators involved in activating endothelial cells. Intriguingly, we also identified different downstream mechanisms regulating endothelial adhesion molecules such as E-selectin, VCAM-1, and ICAM-1. E-selectin is strongly regulated by IL-1 and TNF-α, whereas ICAM-1 and VCAM-1 are driven more by TNF-α. We also found that activated endothelial cells are a major source of IL-6 production, corroborating a previous study (25).

Interestingly, even after blocking IL-1 and TNF-α, we were able to identify strong activation of IFN-α/β and IFN-γ pathways in endothelial cells. Several studies have already identified IFN-α/β pathway in leukocytes in response to bacteria (26) and fungi (27). In endothelial cells, although, IFN-α/β has been shown to promote endothelial proliferation *in vitro* (28), and reduce intracellular NO generation and impair fibrinolysis of HUVECs *in vitro* (29), the precise impact of IFN-α/β on endothelial function in the context of bloodstream infection or sepsis is not clear. Since NO production from the endothelium maintains blood pressure and blood flow (30) and reduced NO bioactivity is associated with sepsis severity (31), it will be relevant to study the effect of neutralizing IFNs to improve sepsis outcomes. In fact, it is currently being discussed whether IFN-β should be neutralized during the hyper-inflammatory phase in sepsis patients due to its contribution to pro-inflammation and/or whether it needs to be supplemented while patients are in the hypo-inflammatory phase given its ability to restore and reverse immunosuppression (32).

Further studies should investigate which inflammatory mediator(s) from activated leukocytes induced IFN-α and IFN-β signaling in endothelial cells. We observed the mRNA expression of IFN-α/β receptors (*IFNAR1, IFNAR2*) in endothelial cells across all the stimulatory pathogens we studied, but could not detect IFN-α or IFN-β in the medium of activated leukocytes (data not shown). Nevertheless, we observed a high amount of IFN- γ released by leukocytes in response to *C. albicans* and also increased RNA expression of IFN- γ receptor (*IFNGR1*) in endothelial cells. We also observed that *C. albicans-*stimulated leukocytes secrete the most potent mix of mediators for inducing endothelial activation, which suggests that *C. albicans* could be a good model to represent the broad impact of leukocyte signals on endothelial cells. One could use it, for example, as a model to study the interaction of leukocytes and endothelial cells with more functional assays to study the biological effect of IFN-activation on endothelial function.

Interestingly, we also observed that *TLR3* (TLR-3)*, DDX58* (RIG-I), and *NLRC5*, together with *IRF-1, -2, -3*, and *-7* were highly expressed in endothelial cells exposed exclusively to *C. albicans-* and *S. pneumoniae*-derived supernatants. RIG-I and IRF-1 are important mediators of endothelial activation in response to LPS and TNF-α as described previously (9, 33, 34). Nevertheless, what regulates the expression of these upstream molecules, either cytosolic DNA or other mediators present in the supernatants, remains elusive.

One of the limitation of our study is the use of heat-killed pathogens, which mounts, to some extent, differences in the host response to pathogens due to the exposure of ligands and the lack of dynamic interactions between the pathogens and the host cells. It has been shown that heat-killed pathogens (such as *Bordetella pertussis*) induce consistently high RNA expression of inflammatory cytokines (such as TNF-α, MIP-1β, IL1-α, and IL-1β) over time, whereas live bacteria induce a transient increase of those genes that was followed by gene suppression (35). Live bacteria, on the other hand, can induce inflammasome activation, altering the amount of secreted IL-1β in macrophages and dendritic cells whereas heat-killed pathogens cannot (36). In our study, although each heat-killed pathogen induced the responses in PBMCs (at RNA levels), which match with the typically known pathogenic toxins and ligands, to what extent the differences between the heat-killed and live pathogens affect the interaction between leukocytes and endothelial cells remains to be further investigated.

Future studies, if possible, should also investigate the host response to live attenuated pathogens or heat-killed pathogen lysate supplemented with bacterial RNA (36). Secondly, although we characterized the global transcription and protein expression levels during leukocyte and endothelial cells cross-talk, follow-up studies are needed to understand the consequences of up-regulated IFN signaling in endothelial cells during sepsis. In addition, it will be important to also study the impact of interaction between neutrophils together with leukocytes and endothelial cells in human-relevant model systems. In conclusion, our work suggests that activation of IFN pathways in endothelial cells plays an important role in the context of sepsis.

## Data Availability Statement

The sequencing data generated by this study was deposited to GEO, access number: GSE131590.

## Ethics Statement

Ethical permission for this study was approved by the Ethical Committee of Radboud University Nijmegen (nr 42561.091.12). The patients/participants provided written informed consent to participate in this study.

## Author Contributions

YL, VK, and JM shared the conceptualization. KL, JM, and VK designed the study and prepared the manuscript. KL, MJ, and JP performed experiments. XC, VM, and YL analyzed the transcriptome data. LJ, MN, SW, and CW provided reagents, protocols, and facilities to conduct stimulation/blocking experiments. LJ, MN, CW, YL, JM, and VK interpreted results and critically assessed the manuscript.

### Conflict of Interest

The authors declare that the research was conducted in the absence of any commercial or financial relationships that could be construed as a potential conflict of interest.
