# Supplementary material for: Leukocyte-Released Mediators in Response to Both Bacterial and Fungal Infections Trigger IFN Pathways, Independent of IL-1 and TNF-α, in Endothelial Cells
Source: Front Immunol. 2019 Oct 25;10:2508. doi: 10.3389/fimmu.2019.02508 (PMC6824321; doi:10.3389/fimmu.2019.02508)
Supplement: Supplementary file 1 [file Data_Sheet_1.docx]

**SUPPLEMENTAL DATA**

**Supplemental figure S1**

**
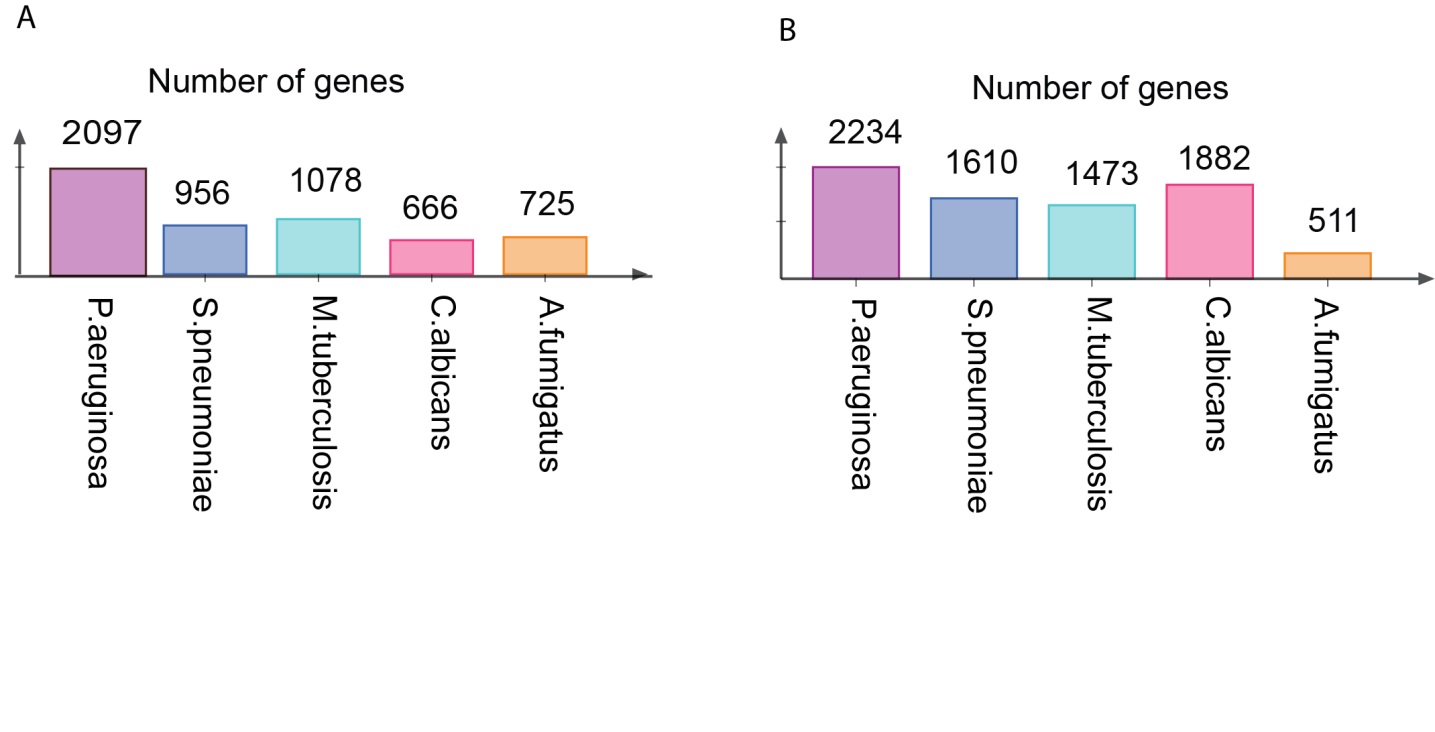
**

**S1 Fig. Number of genes differentially expressed in PBMCs upon exposure to various stimulation**. PBMC transcriptome analyzed after (A) 4 hours and (B) 24 hours of stimulation.

**Supplemental figure S2**


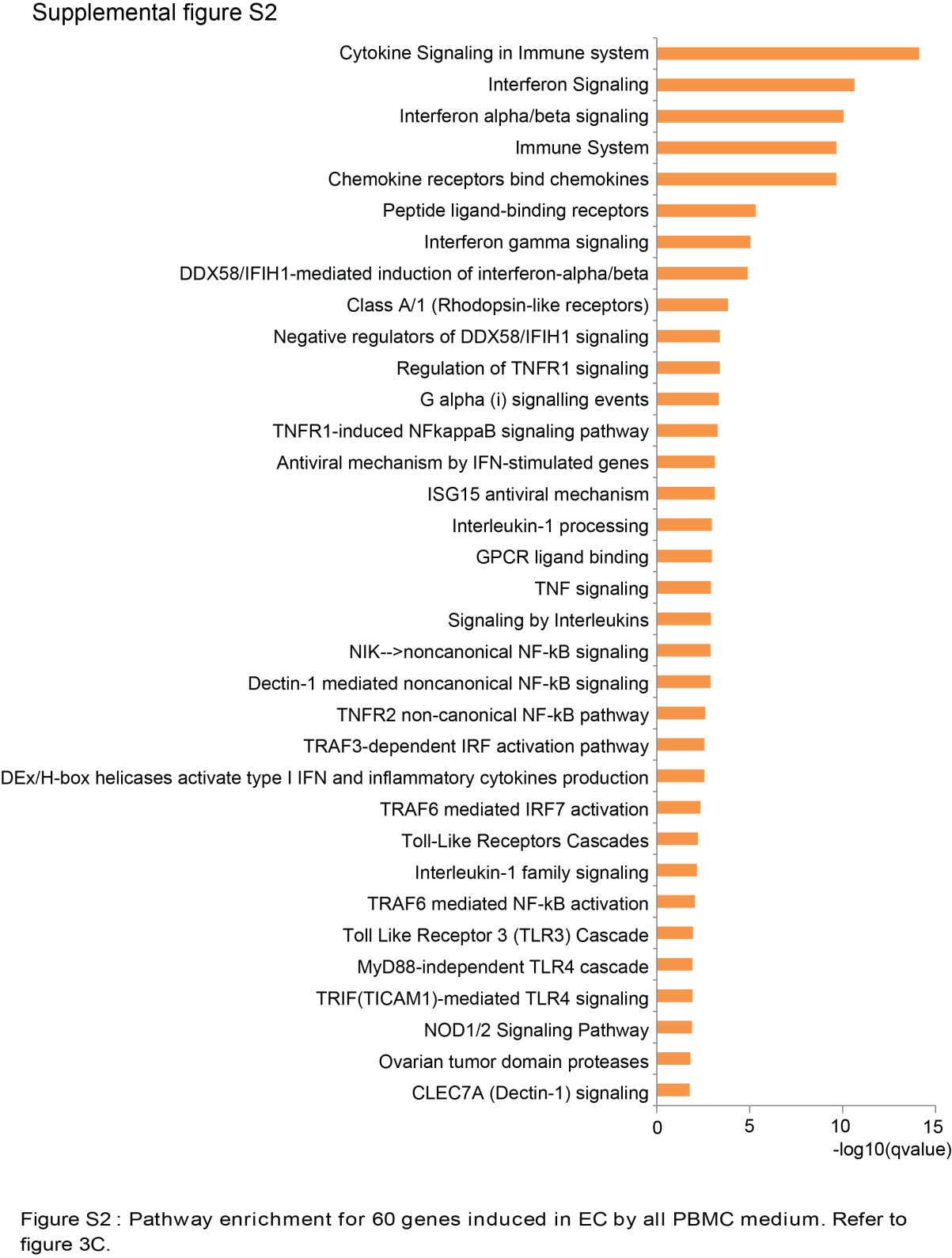


**S2 Fig. Pathway enrichment for common genes induced in EC by all PBMC-released mediators.** Among 65 common genes responding to PBMC-released mediators (refer to Fig 3C), 5 genes are also activated by direct stimulation of EC with LPS, *S.pneumoniae* and *C.albicans*. Pathway enrichment was perform for the unique 60/65 genes are activated only by PBMC medium. x-axis: enrichment score (-log10 of qvalue), y-axis: names of Reactome pathway.

**Supplemental figure S3**


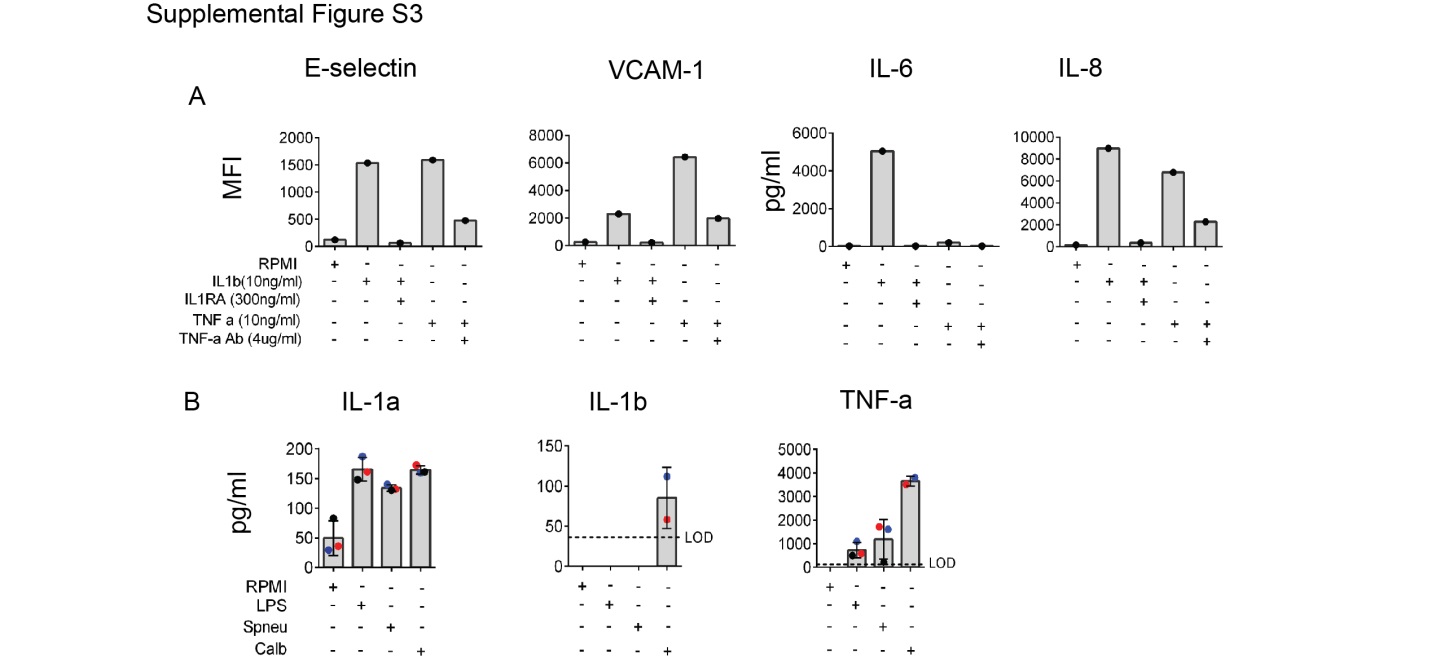


**S3 Fig.** **Validation of blocking doses of IL1RA and TNF-α Ab used**. (A) Protein expression levels of activating markers on ECs (E-selectin, VCAM-1, IL-6 and IL-8) upon direct stimulation of IL1 & TNF-α versus IL1+ IL1RA & TNF-α+ TNF-α Ab. (B) Concentration of IL-1α , IL-1β and TNF-α in PBMC supernatants measured by ELISA. Colors coordinate to donors. Data are represented for 2 independent experiments.

**Supplemental table S1**

| **Condition** | **Gene group** | **Reactome pathways** | **-log10**  **(qValue)** |
| --- | --- | --- | --- |
| P.aeruginosa_4h | Upregulated genes | DDX58/IFIH1-mediated induction of interferon-alpha/beta | 1,28 |
| P.aeruginosa_4h | Upregulated genes | PTK6 Activates STAT3 | 1,26 |
| P.aeruginosa_4h | Upregulated genes | Interleukin-1 family signaling | 1,23 |
| P.aeruginosa_4h | Upregulated genes | Homologous DNA Pairing and Strand Exchange | 1,19 |
| P.aeruginosa_4h | Upregulated genes | Postsynaptic nicotinic acetylcholine receptors | 1,18 |
| P.aeruginosa_4h | Upregulated genes | Activation of Nicotinic Acetylcholine Receptors | 1,18 |
| P.aeruginosa_4h | Upregulated genes | Acetylcholine binding and downstream events | 1,18 |
| P.aeruginosa_4h | Upregulated genes | Keratan sulfate biosynthesis | 1,14 |
| P.aeruginosa_4h | Upregulated genes | SMAC binds to IAPs | 1,14 |
| P.aeruginosa_4h | Upregulated genes | SMAC-mediated dissociation of IAP:caspase complexes | 1,14 |
| P.aeruginosa_4h | Upregulated genes | SMAC-mediated apoptotic response | 1,14 |
| P.aeruginosa_4h | Upregulated genes | FasL/ CD95L signaling | 1,14 |
| P.aeruginosa_4h | Upregulated genes | Interleukin-18 signaling | 1,14 |
| P.aeruginosa_4h | Upregulated genes | Ligand-dependent caspase activation | 1,07 |
| P.aeruginosa_4h | Upregulated genes | Sema3A PAK dependent Axon repulsion | 1,07 |
| P.aeruginosa_4h | Suppressed genes | G alpha (q) signalling events | 1,78 |
| P.aeruginosa_4h | Suppressed genes | Arachidonate production from DAG | 1,59 |
| P.aeruginosa_4h | Suppressed genes | Neuronal System | 1,33 |
| P.aeruginosa_24h | Upregulated genes | Cytokine Signaling in Immune system | 5,90 |
| P.aeruginosa_24h | Upregulated genes | Programmed Cell Death | 3,99 |
| P.aeruginosa_24h | Upregulated genes | Apoptosis | 3,30 |
| P.aeruginosa_24h | Upregulated genes | TNFR2 non-canonical NF-kB pathway | 3,02 |
| P.aeruginosa_24h | Upregulated genes | Interleukin-1 processing | 2,77 |
| P.aeruginosa_24h | Upregulated genes | TNF signaling | 2,74 |
| P.aeruginosa_24h | Upregulated genes | Intrinsic Pathway for Apoptosis | 2,49 |
| P.aeruginosa_24h | Upregulated genes | RIP-mediated NFkB activation via ZBP1 | 2,49 |
| P.aeruginosa_24h | Upregulated genes | Regulation of TNFR1 signaling | 2,49 |
| P.aeruginosa_24h | Upregulated genes | Death Receptor Signalling | 2,39 |
| P.aeruginosa_24h | Upregulated genes | Immune System | 2,36 |
| P.aeruginosa_24h | Upregulated genes | TNFR1-induced NFkappaB signaling pathway | 2,36 |
| P.aeruginosa_24h | Upregulated genes | ZBP1(DAI) mediated induction of type I IFNs | 2,35 |
| P.aeruginosa_24h | Upregulated genes | TNFs bind their physiological receptors | 2,24 |
| P.aeruginosa_24h | Upregulated genes | NOD1/2 Signaling Pathway | 2,07 |
| P.aeruginosa_24h | Upregulated genes | Toll Like Receptor 4 (TLR4) Cascade | 2,04 |
| P.aeruginosa_24h | Upregulated genes | RIPK1-mediated regulated necrosis | 1,98 |
| P.aeruginosa_24h | Upregulated genes | Regulated Necrosis | 1,98 |
| P.aeruginosa_24h | Upregulated genes | Toll Like Receptor 3 (TLR3) Cascade | 1,95 |
| P.aeruginosa_24h | Upregulated genes | TRIF(TICAM1)-mediated TLR4 signaling | 1,91 |
| P.aeruginosa_24h | Upregulated genes | MyD88-independent TLR4 cascade | 1,91 |
| P.aeruginosa_24h | Upregulated genes | SMAC binds to IAPs | 1,87 |
| P.aeruginosa_24h | Upregulated genes | SMAC-mediated dissociation of IAP:caspase complexes | 1,87 |
| P.aeruginosa_24h | Upregulated genes | SMAC-mediated apoptotic response | 1,87 |
| P.aeruginosa_24h | Upregulated genes | TP53 Regulates Transcription of Genes Involved in Cytochrome C Release | 1,84 |
| P.aeruginosa_24h | Upregulated genes | TP53 Regulates Transcription of Cell Death Genes | 1,82 |
| P.aeruginosa_24h | Upregulated genes | Toll-Like Receptors Cascades | 1,75 |
| P.aeruginosa_24h | Upregulated genes | Signaling by Interleukins | 1,69 |
| P.aeruginosa_24h | Upregulated genes | Cytosolic sensors of pathogen-associated DNA | 1,67 |
| P.aeruginosa_24h | Upregulated genes | Apoptotic factor-mediated response | 1,67 |
| P.aeruginosa_24h | Upregulated genes | Caspase activation via extrinsic apoptotic signalling pathway | 1,62 |
| P.aeruginosa_24h | Upregulated genes | TAK1 activates NFkB by phosphorylation and activation of IKKs complex | 1,47 |
| P.aeruginosa_24h | Upregulated genes | Apoptotic cleavage of cell adhesion proteins | 1,42 |
| P.aeruginosa_24h | Upregulated genes | Regulated proteolysis of p75NTR | 1,42 |
| P.aeruginosa_24h | Upregulated genes | Interferon Signaling | 1,35 |
| P.aeruginosa_24h | Upregulated genes | Ovarian tumor domain proteases | 1,35 |
| P.aeruginosa_24h | Upregulated genes | Apoptotic cleavage of cellular proteins | 1,35 |
| P.aeruginosa_24h | Upregulated genes | DEx/H-box helicases activate type I IFN and inflammatory cytokines production | 1,34 |
| P.aeruginosa_24h | Suppressed genes | Extracellular matrix organization | 2,41 |
| P.aeruginosa_24h | Suppressed genes | Integrin cell surface interactions | 1,92 |
| P.aeruginosa_24h | Suppressed genes | Signaling by MST1 | 1,63 |
| P.aeruginosa_24h | Suppressed genes | Neutrophil degranulation | 1,63 |
| P.aeruginosa_24h | Suppressed genes | O-glycosylation of TSR domain-containing proteins | 1,58 |
| P.aeruginosa_24h | Suppressed genes | Collagen biosynthesis and modifying enzymes | 1,50 |
| P.aeruginosa_24h | Suppressed genes | Choline catabolism | 1,50 |
| P.aeruginosa_24h | Suppressed genes | Glycerophospholipid catabolism | 1,37 |
| S.pneumoniae_4h | Upregulated genes | Interferon gamma signaling | 1,54 |
| S.pneumoniae_4h | Suppressed genes | CRMPs in Sema3A signaling | 2,35 |
| S.pneumoniae_4h | Suppressed genes | Cytokine Signaling in Immune system | 1,84 |
| S.pneumoniae_4h | Suppressed genes | Semaphorin interactions | 1,84 |
| S.pneumoniae_4h | Suppressed genes | Signaling by Interleukins | 1,84 |
| S.pneumoniae_4h | Suppressed genes | Immune System | 1,84 |
| S.pneumoniae_4h | Suppressed genes | Phase I - Functionalization of compounds | 1,70 |
| C.albicans_24h | Upregulated genes | Antigen processing-Cross presentation | 2,06 |
| C.albicans_24h | Upregulated genes | Reuptake of GABA | 2,06 |
| C.albicans_24h | Upregulated genes | Cross-presentation of particulate exogenous antigens (phagosomes) | 1,70 |
| C.albicans_24h | Upregulated genes | TRIF-mediated programmed cell death | 1,69 |
| C.albicans_24h | Upregulated genes | Immune System | 1,69 |
| C.albicans_24h | Upregulated genes | Transport of small molecules | 1,69 |
| C.albicans_24h | Upregulated genes | TRAF6-mediated induction of TAK1 complex within TLR4 complex | 1,68 |
| C.albicans_24h | Upregulated genes | G alpha (i) signalling events | 1,66 |
| C.albicans_24h | Upregulated genes | Glycosphingolipid metabolism | 1,66 |
| C.albicans_24h | Upregulated genes | RHO GTPases Activate NADPH Oxidases | 1,66 |
| C.albicans_24h | Upregulated genes | Activation of IRF3/IRF7 mediated by TBK1/IKK epsilon | 1,66 |
| C.albicans_24h | Upregulated genes | Chemokine receptors bind chemokines | 1,59 |
| C.albicans_24h | Upregulated genes | Regulation of TLR by endogenous ligand | 1,55 |
| C.albicans_24h | Upregulated genes | Ligand-dependent caspase activation | 1,55 |
| C.albicans_24h | Upregulated genes | Naﰩ dependent neurotransmitter transporters | 1,49 |
| C.albicans_24h | Upregulated genes | The canonical retinoid cycle in rods (twilight vision) | 1,42 |
| C.albicans_24h | Upregulated genes | ER-Phagosome pathway | 1,36 |
| C.albicans_24h | Upregulated genes | IKK complex recruitment mediated by RIP1 | 1,33 |
| C.albicans_24h | Upregulated genes | Other interleukin signaling | 1,33 |
| C.albicans_24h | Upregulated genes | Caspase activation via extrinsic apoptotic signalling pathway | 1,33 |

**S1 Table. Significant pathways enriched by pathogen-specific gene sets in PBMCs**. PBMCs were stimulated with three types of pathogens (*P.aeruginosa*, *S.pneumoniae* and *C.albicans* at 2 time points (4 hours and 24 hours) (refer to Fig 1C and Fig 1E). Differentially expressed genes upon each condition were classified into two classes, Upregulated genes and Suppressed genes, in relatives to their expression level in control samples. Reactome pathway analysis were performed for each set of genes. Values were shown as –log10(qvalue).
