# Supplementary figures and images for: Leukocyte-Released Mediators in Response to Both Bacterial and Fungal Infections Trigger IFN Pathways, Independent of IL-1 and TNF-α, in Endothelial Cells
Source: Front Immunol. 2019 Oct 25;10:2508. doi: 10.3389/fimmu.2019.02508 (PMC6824321; doi:10.3389/fimmu.2019.02508)

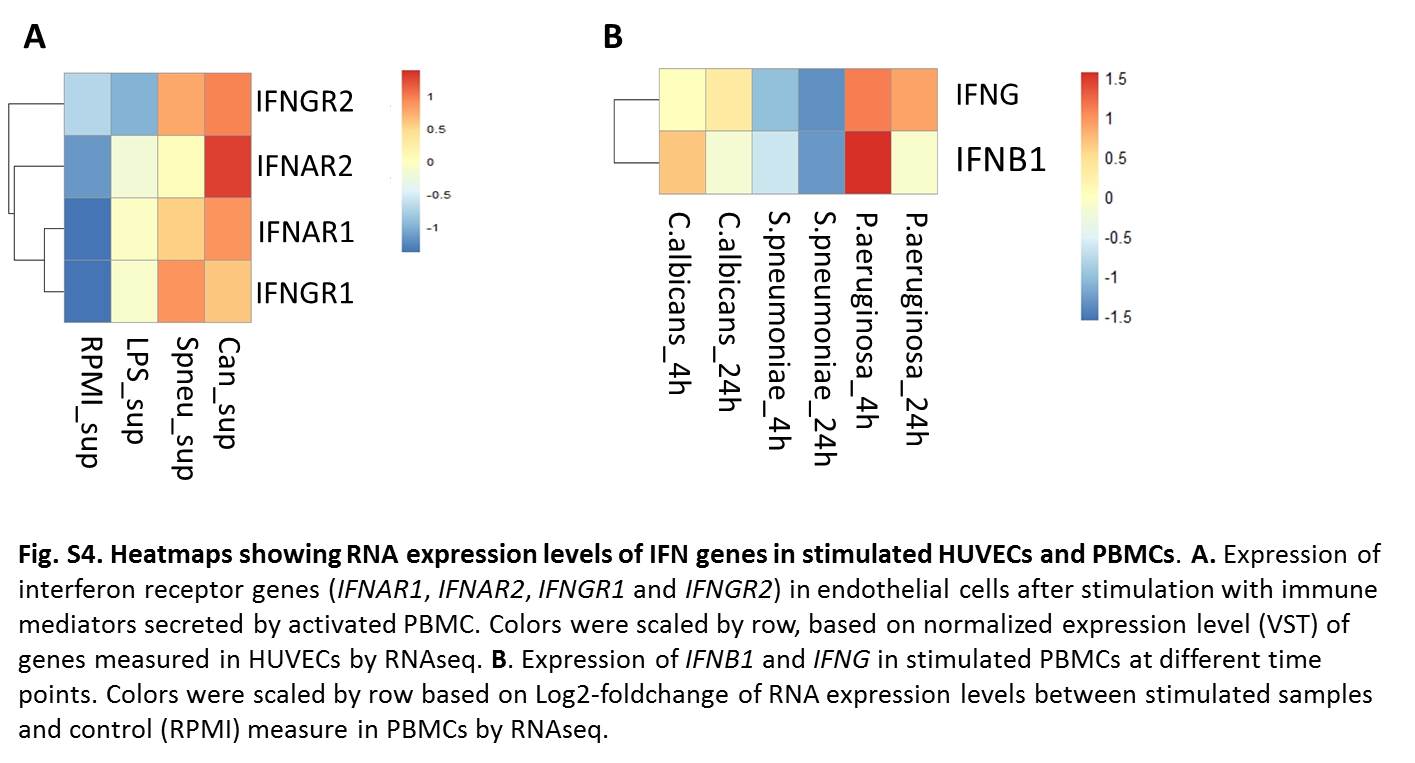

Supplement: Supplementary file 2 [file Image_4.jpeg]
